# Ascites, Pleural, and Pericardial Effusion in Primary Hypothyroidism: A Rare Case Report

**DOI:** 10.7759/cureus.50429

**Published:** 2023-12-13

**Authors:** Pedro Gomes Santos, Roberto Calças Marques, Pedro Martins dos Santos, Catarina Carreira da Costa, Mihail Mogildea

**Affiliations:** 1 Internal Medicine, Centro Hospitalar Universitário do Algarve - Hospital de Faro, Faro, PRT; 2 Nephrology, Centro Hospitalar Universitário do Algarve - Hospital de Faro, Faro, PRT

**Keywords:** hypothyroidism, pericardial effusion, pleural effusion, ascites, case report, hashimoto’s thyroiditis

## Abstract

Hypothyroidism is caused by a deficiency of thyroid hormones and is a common endocrine disorder worldwide. It can affect nearly every organ, resulting in multiple clinical manifestations. Ascites, pleural effusion, and pericardial effusion, although less frequent than peripheral edema, can also be present. These manifestations are thought to be caused by increased vascular permeability to albumin, extravasation of mucopolysaccharides, and inappropriate antidiuretic hormone secretion. Most effusions in hypothyroid patients resolve with thyroxine replacement therapy. However, due to the insidious and nonspecific nature of these symptoms, hypothyroidism is seldom considered a differential diagnosis. We report a case of a 48-year-old male with pericardial effusion, pleural effusion, and ascites due to primary hypothyroidism. Although isolated effusions can be frequent in patients with hypothyroidism, the presentation of Hashimoto's thyroiditis as a combination of pericardial effusion, pleural effusion, and ascites is extremely rare. With this case report, we highlight the importance of considering hypothyroidism as a possible cause of unexplained polyserositis, even in the absence of other signs and symptoms.

## Introduction

Primary hypothyroidism is a common endocrine disorder that can impact between 4% to 10% of the overall population. It is characterized by insufficient levels of thyroid hormones, and the primary cause of this condition is Hashimoto's thyroiditis, an autoimmune disease [1].

Low thyroid hormone levels can have an impact on nearly all organs, resulting in various clinical manifestations. Individuals with hypothyroidism often experience peripheral edema as a typical symptom, while ascites, pleural effusion, and pericardial effusion are less frequently observed [2-4]. The mechanism behind the formation of edema and effusions is not fully understood. It is believed to result from the absence of thyroid hormones, which can either trigger the release of histamine by mast cells or exert a direct effect on the capillary's endothelial layers [5,6]. Ultimately, this process leads to increased vascular permeability to albumin [3]. Extravasation of mucopolysaccharides into body cavities and inappropriate antidiuretic hormone secretion may also play a role [2,7].

Here, we report a case of Hashimoto's thyroiditis with an uncommon presentation, contributing to the understanding of this condition.

## Case presentation

A 48-year-old male was electively admitted to the internal medicine ward for the evaluation of bilateral pleural effusion. He had a medical history of arterial hypertension, chronic heart failure due to a myocardial infarction four years prior, persecutory delusion, and end-stage kidney failure secondary to chronic glomerulonephritis with 20 years of diagnosis. He had been undergoing hemodialysis for the previous four years following kidney allograft failure from a deceased donor.

**Figure 1:**
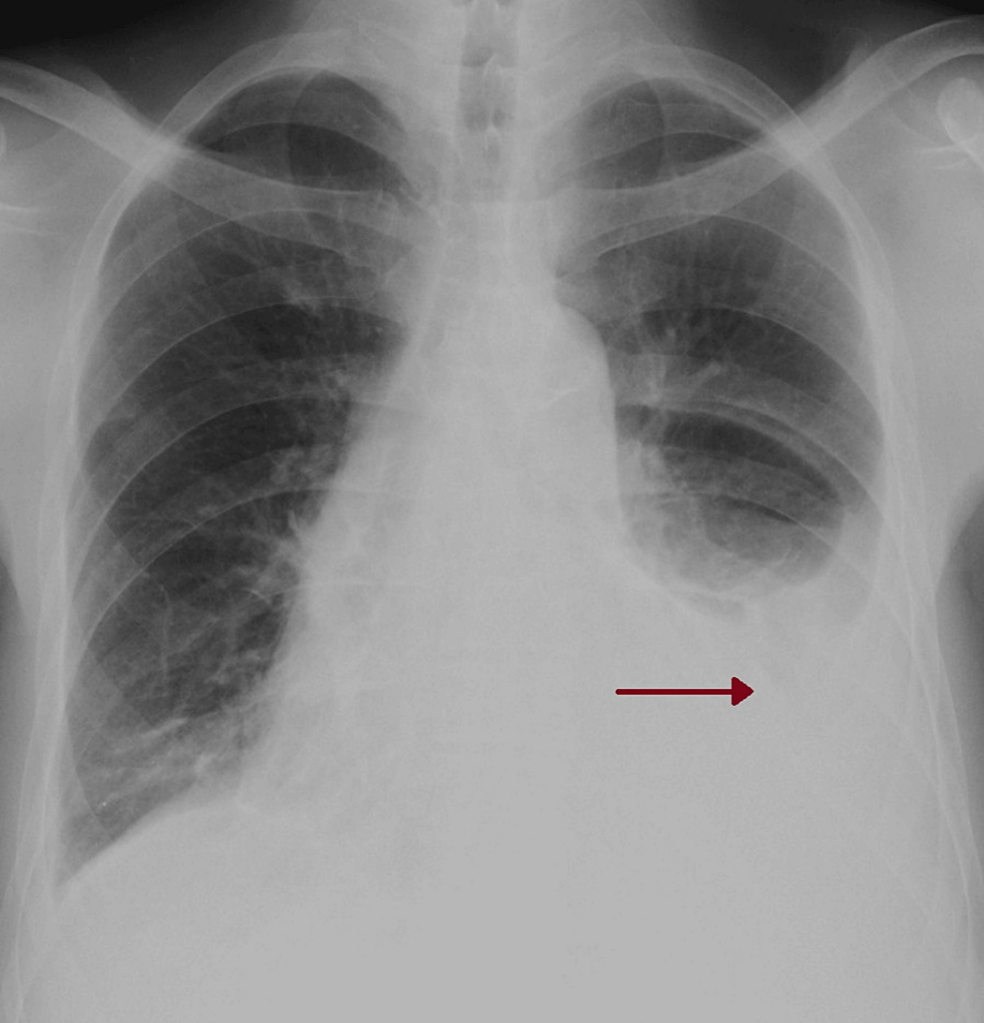
First chest x-ray at a hemodialysis clinic Red arrow: pleural effusion

Four months before hospital admission, the patient presented to the hemodialysis clinic with anhedonia and a nocturnal dry cough, without any other symptoms. A chest x-ray revealed a left pleural effusion (Figure 1). He was diagnosed with acute tracheobronchitis and volume overload and was treated with amoxicillin/clavulanic acid 500/125mg every 12 hours for seven days, along with azithromycin 500mg once daily for five days. Additionally, the patient's dry weight was reduced.

**Figure 2:**
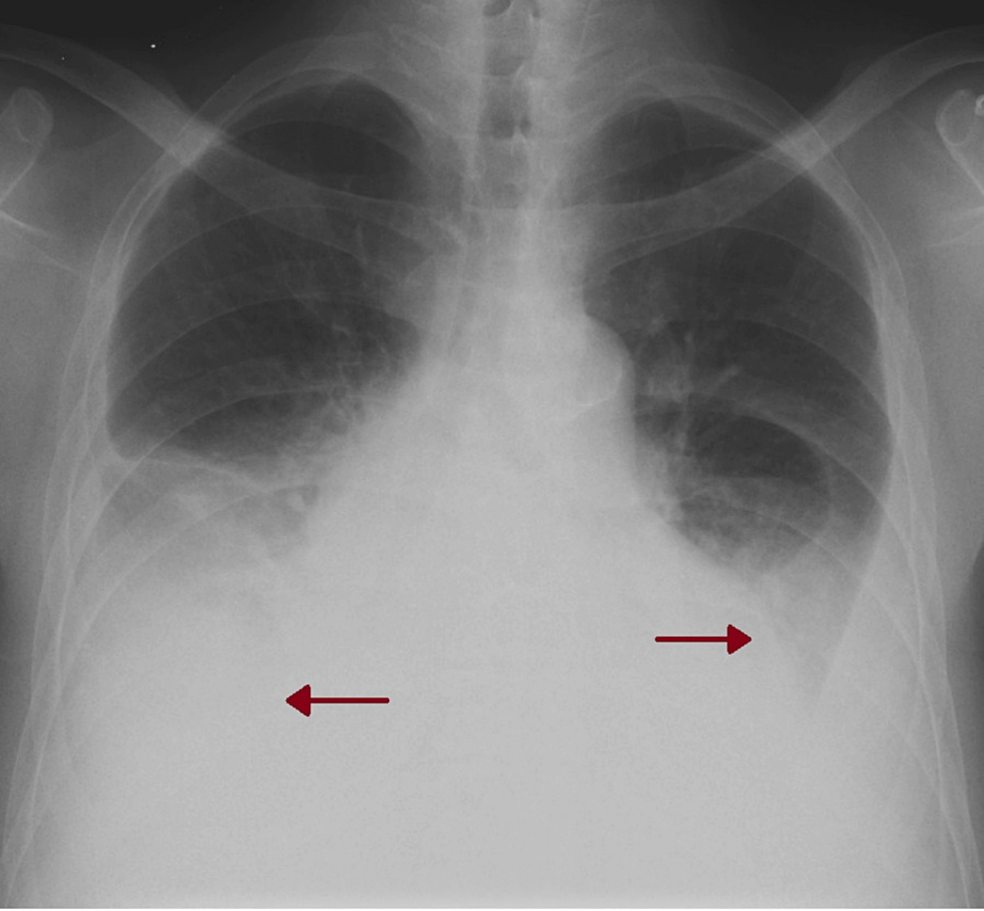
Second chest x-ray at a hemodialysis clinic (after two months) Red arrows: bilateral pleural effusion

**Figure 3:**
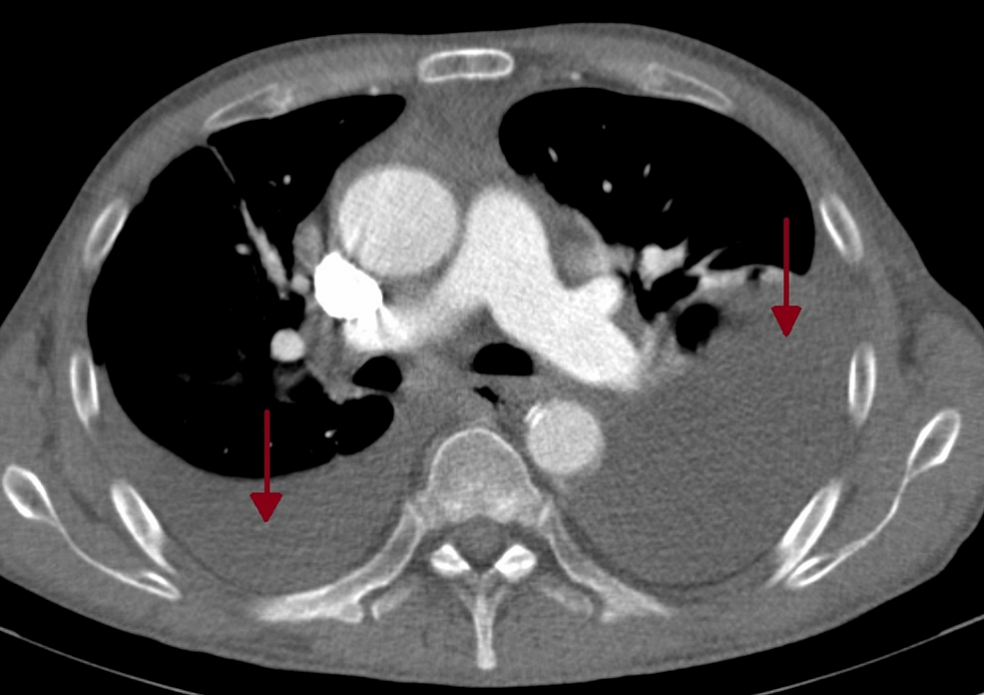
Chest computed tomography Red arrows: bilateral pleural effusion

**Figure 4:**
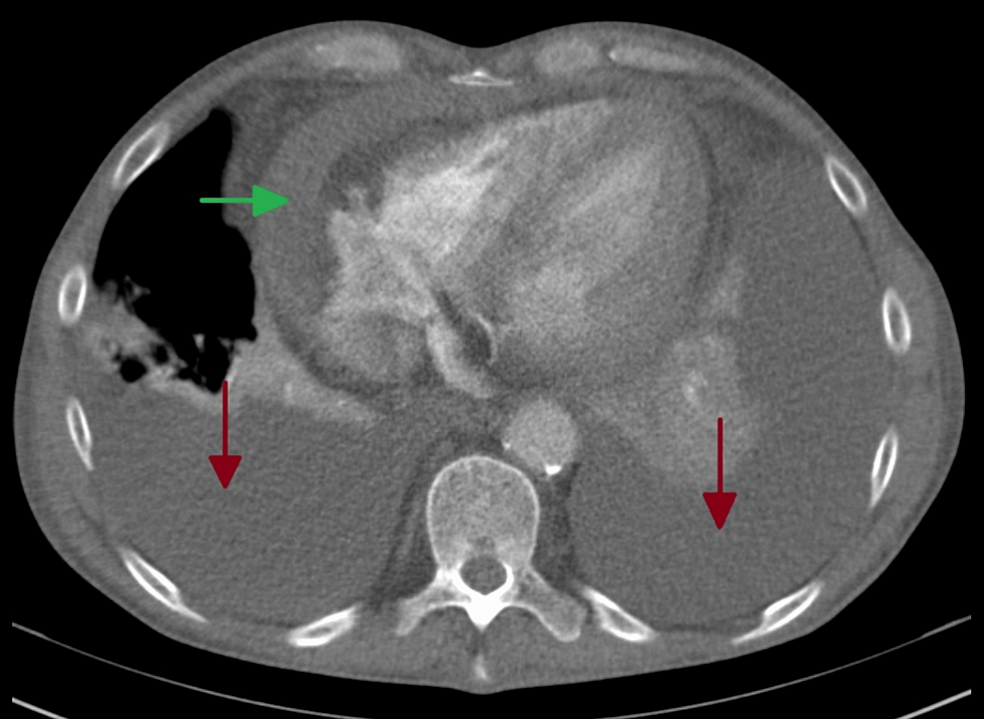
Chest computed tomography Red arrows: bilateral pleural effusion; green arrow: pericardial effusion

After two months of no symptomatic improvement, a chest x-ray revealed bilateral pleural effusion (Figure 2). As a result, the patient was prescribed levofloxacin 500mg every 48 hours for seven days and referred to a pneumology consultation, where thoracocentesis and pleural biopsies were performed. No neoplastic cells were found in the pleural fluid, and cultures yielded negative results. Additionally, the pleural histology showed no abnormalities. Based on Light’s criteria, the effusion was classified as exudative (Table 1) [8]. A chest computed tomography (CT) scan revealed a moderate bilateral pleural effusion in the organizing stage on the left, along with a small pericardial effusion (Figures 3, 4). The antinuclear antibody (ANA) determination was negative, but there was an elevation in anti-dsDNA antibody levels (Table 1).

**Table 1:** Laboratory investigations

Tested fluid	Parameter	Value at diagnosis	Normal range
Blood	Hemoglobin (HGB)	110 g/L	130-170 g/L
	Mean cell hemoglobin concentration (MCHC)	335 g/L	315-345 g/L
	Mean cell volume (MCV)	85.2 fL	83-101 fL
	White blood cells (WBC)	6.9 × 10^9^/L	4-10 × 10^9^/L
	Platelet count	270 × 10^9^/L	150-400 × 10^9^/L
	Total proteins	6.2 g/dL	6.4-8.3 g/dL
	Lactate dehydrogenase (LDH)	159 UI/L	125-243 UI/L
	Antinuclear antibodies (ANA)	Negative	
	Anti-dsDNA antibodies	92 U/mL	<20 U/mL
	Free thyroxin (FT4)	0.47 ng/dL	0.52-3.88 ng/dL
	Thyroid stimulating hormone (TSH )	>100 µUI/mL	0.35-4.94 µUI/mL
	Antithyroglobulin antibody (TgAb)	2841 UI/mL	<150 UI/mL
	Anti-thyroid peroxidase antibody (anti-TPO)	731 UI/mL	<50 UI/mL
Pleural fluid	Total proteins	3 g/dL	1-2 g/dL
	Lactate dehydrogenase (LDH)	116 UI/L	

After the nephrologist's referral, the patient was electively admitted to the internal medicine ward for further evaluation. On admission, heart rate was 75 bpm and regular, blood pressure was 130/75 mmHg, and transcutaneous oxygen saturation was 97% without supplementary oxygen. Auscultation revealed a bilateral absence of breathing sounds in the lower lung fields, and heart sounds were rhythmic and regular, with normal intensity. The patient also exhibited non-pitting edema in the lower extremities. The physical examination did not reveal any other abnormalities.

Additional tests were ordered to elucidate the cause of polyserositis and peripheral edema. A complete blood count revealed normocytic normochromic anemia, with a normal white blood cell (WBC) count and platelet count (Table 1). The echocardiogram demonstrated concentric left ventricular hypertrophy with a wall thickness of 14 mm, suggestive of longstanding hypertension. Pericardial thickening and a small pericardial effusion measuring less than 5 mm were also observed. The abdominal and pelvic CT scans revealed a low volume of ascites and bilateral renal atrophy, with no other abnormalities detected. The patient declined to undergo a colonoscopy and an upper endoscopy to investigate and rule out paraneoplastic polyserositis. The comprehensive diagnostic workup revealed elevated levels of thyroid stimulating hormone (TSH), low levels of free thyroxine (FT4), and high levels of antithyroglobulin antibody (TgAb) and anti-thyroid peroxidase antibody (anti-TPO) (Table 1). These findings were compatible with Hashimoto's thyroiditis, and the patient was initiated on a low dose of levothyroxine (12.5 μg/day p.o.), adjusted for his comorbidities, with a progressive increase.

During hospitalization, the patient developed nosocomial pneumonia with respiratory failure, requiring treatment with broad-spectrum antibiotherapy. The patient was eventually discharged with a prescribed levothyroxine dose of 50 μg/day p.o. and was referred to an internal medicine consultation for further management and follow-up.

**Figure 5:**
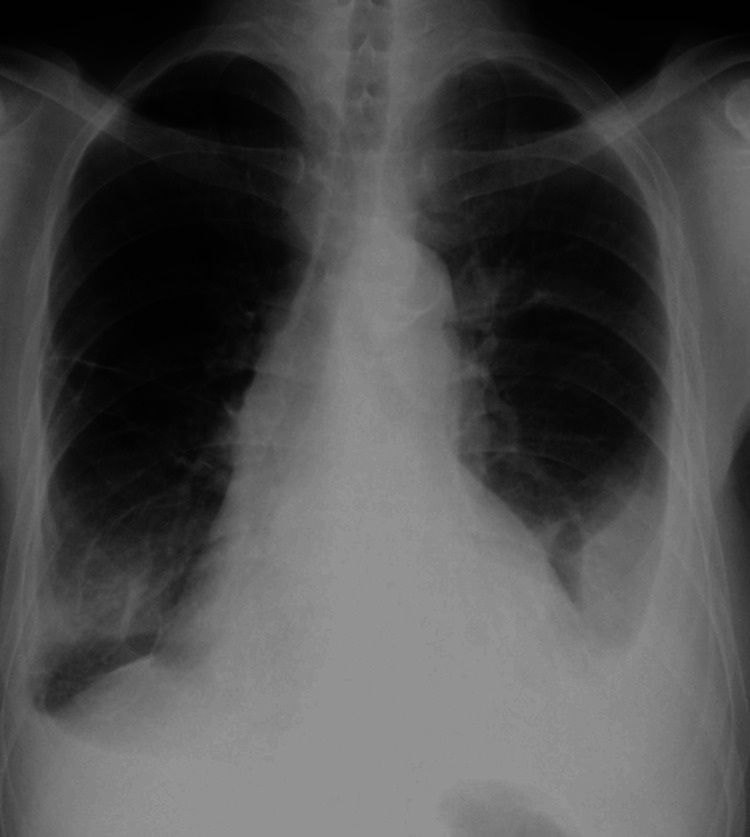
Chest x-ray seven months after hospital discharge

Although the patient missed the internal medicine consultation, follow-up was still ensured by nephrologists at the hemodialysis clinic. After seven months, the patient reported significant improvement in all symptoms, including anhedonia. Upon physical examination, pulmonary auscultation was normal, and there was a complete resolution of edema in the lower extremities. Despite the high TSH values (17.5 µUI/mL), which could be attributed to low compliance with medication, the free thyroxine (FT4) levels were within the normal range (1.05 ng/dL). Additionally, a significant improvement in the pleural effusion was observed on the chest x-ray (Figure 5).

## Discussion

Hypothyroidism is a prevalent endocrine disease worldwide. Despite being detectable through straightforward laboratory tests, it may exhibit a variety of nonspecific symptoms. This can lead to a delayed diagnosis or even go unnoticed if the patient is asymptomatic [6]. In hypothyroidism, tissue edema is frequently observed, while polyserositis is less common. These clinical manifestations are associated with increased vascular permeability to albumin, the leakage of mucopolysaccharides, and the inappropriate secretion of antidiuretic hormone [6,7].

Although pleural effusion in hypothyroidism has been reported with a frequency ranging from 10% to 30%, it is likely underestimated as it is typically of small volume and of minor clinical significance [9]. This manifestation is usually associated with the duration of hypothyroidism rather than its chemical degree [10]. The fluid in pleural effusions associated with hypothyroidism is commonly exudative, but it may exhibit characteristics that are intermediate between transudates and exudates [2,10]. Although exudative pleural effusions are frequently observed in hypothyroidism, the large volume in this patient is relatively uncommon. However, thyroxine replacement therapy is still expected to resolve the effusion [2].

Pericardial effusion is a well-known complication of hypothyroidism, with an incidence ranging from 3% to 37% [3]. In most cases, thyroxine replacement is the only treatment needed for pericardial effusions. However, in cases of cardiac tamponade, urgent pericardiocentesis is mandatory [6]. Our patient presented with only a small pericardial effusion.

On the contrary, myxedema ascites is a rare complication of hypothyroidism, occurring in only 4% of patients and accounting for less than 1% of all cases of ascites [11]. The serum-ascites albumin gradient (SAAG) is usually high (≥ 1.1 g/dL) with a high fluid protein level (>2.5 g/dL), but cases of myxedema ascites with low SAAG have also been reported [4,11,12]. Despite the recommendation for diagnostic paracentesis in cases of new-onset ascites, it was not possible for our patient due to the small volume of ascites.

Although the patient's hypothyroidism resulted from Hashimoto's thyroiditis, an autoimmune disorder, polyserositis in hypothyroidism does not seem to have an autoimmune explanation. In fact, effusions have also been reported in cases of hypothyroidism caused by iodine deficiency and postsurgical hypothyroidism [2,3]. Achieving a euthyroid state is the definitive treatment for this condition [3]. An increase in anti-dsDNA antibody levels can occur in up to 12% of patients with Hashimoto’s thyroiditis [13]. However, in the presence of negative antinuclear antibodies (ANA), the elevation of anti-dsDNA antibody levels in our patient was likely due to a false positive in the ELISA assay. Even in the absence of other autoantibodies besides TgAb and anti-TPO, patients with Hashimoto’s thyroiditis are at risk of developing polyautoimmunity, defined by the coexistence of two or more autoimmune diseases [13].

Isolated effusions in natural body cavities can be frequent in patients with hypothyroidism. However, the presentation of Hashimoto's thyroiditis as a combination of pericardial effusion, pleural effusion, and ascites is extremely rare. Due to this unusual presentation, our differential diagnosis included various etiologies such as autoimmune, autoinflammatory, infectious, metabolic, toxic origin, or neoplastic diseases. Paraneoplastic polyserositis, which accounts for up to 20% of cases, was not fully investigated due to the patient's refusal to undergo endoscopic exams [14]. Nonetheless, given the complete resolution of pleural effusion following hormone replacement therapy, other hypotheses were considered less likely.

## Conclusions

Hypothyroidism is a prevalent condition worldwide, and its symptoms can be nonspecific and gradual. However, the diagnosis is simple and easily accessible, and most effusions seen in hypothyroid patients typically resolve with thyroxine replacement therapy. Therefore, it is important to consider hypothyroidism in patients with unexplained pericardial effusion, pleural effusion, or ascites, regardless of other signs and symptoms. With this case report, we aim to raise awareness of this uncommon manifestation, potentially leading to earlier diagnosis and management.
